# Integrated stepped alcohol treatment for patients with HIV and at-risk alcohol use: a randomized trial

**DOI:** 10.1186/s13722-020-00200-y

**Published:** 2020-07-29

**Authors:** E. Jennifer Edelman, Stephen A. Maisto, Nathan B. Hansen, Christopher J. Cutter, James Dziura, Yanhong Deng, Lynn E. Fiellin, Patrick G. O’Connor, Roger Bedimo, Cynthia L. Gibert, Vincent C. Marconi, David Rimland, Maria C. Rodriguez-Barradas, Michael S. Simberkoff, Janet P. Tate, Amy C. Justice, Kendall J. Bryant, David A. Fiellin

**Affiliations:** 1 Yale School of Medicine, 367 Cedar Street, ESH A, New Haven, CT 06510 USA; 2 Center for Interdisciplinary Research on AIDS, Yale School of Public Health, New Haven, CT 06510 USA; 3 Syracuse University, Syracuse, NY 13244 USA; 4 College of Public Health, University of Georgia, Athens, GA 30602 USA; 5 Yale Center for Analytic Sciences, Yale University School of Public Health, New Haven, CT 06511 USA; 6 Veterans Affairs North Texas Health Care System and UT Southwestern, Dallas, TX 75216 USA; 7 D.C. VAMC and George, Washington University School of Medicine and Health Sciences, Washington, D.C 20422 USA; 8 Atlanta VAMC and Emory University School of Medicine, Atlanta, GA 30033 USA; 9 Michael E. DeBakey VAMC and Baylor College of Medicine, Texas Houston, Houston, TX 77030 USA; 10 VA NY Harbor Healthcare System and New York University School of Medicine, New York, NY 10010 USA; 11 VA Connecticut Healthcare System, Veterans Aging Cohort Study, West Haven, CT 06516 USA; 12 National Institute On Alcohol Abuse and Alcoholism HIV/AIDS Program, Bethesda, MD 20892-7003 USA

**Keywords:** HIV, Alcohol-related disorders, Delivery of health care, Integrated

## Abstract

**Background:**

At-risk levels of alcohol use threaten the health of patients with HIV (PWH), yet evidence-based strategies to decrease alcohol use and improve HIV-related outcomes in this population are lacking. We examined the effectiveness of integrated stepped alcohol treatment (ISAT) on alcohol use and HIV outcomes among PWH and at-risk alcohol use.

**Methods:**

In this multi-site, randomized trial conducted between January 28, 2013 through July 14, 2017, we enrolled PWH and at-risk alcohol use [defined as alcohol consumption of ≥ 14 drinks per week or ≥ 4 drinks per occasion in men ≤ 65 years old or ≥ 7 drinks per week or ≥ 3 drinks per occasion in women or men > 65 years old]. ISAT (n = 46) involved: Step 1- Brief Negotiated Interview with telephone booster, Step 2- Motivational Enhancement Therapy, and Step 3- Addiction Physician Management. Treatment as usual (TAU) (n = 47) involved receipt of a health handout plus routine care. Analyses were conducted based on intention to treat principles.

**Results:**

Despite a multi-pronged approach, we only recruited 37% of the target population (n = 93/254). Among ISAT participants, 50% advanced to Step 2, among whom 57% advanced to Step 3. Participants randomized to ISAT and TAU had no observed difference in drinks per week over the past 30 days at week 24 (primary outcome) [least square means (Ls mean) (95% CI) = 8.8 vs. 10.6; adjusted mean difference (AMD) (95% CI) =  − 0.4 (− 3.9, 3.0)].

**Conclusion:**

An insufficient number of patients were interested in participating in the trial. Efforts to enhance motivation of PWH with at-risk alcohol use to engage in alcohol-related research and build upon ISAT are needed.

*Trial registration* Clinicaltrials.gov: NCT01410123, First posted August 4, 2011

## Background

At-risk alcohol use, defined as alcohol consumption of ≥ 14 drinks per week or ≥ 4 drinks per occasion in men ≤ 65 years old or ≥ 7 drinks per week or ≥ 3 drinks per occasion in women or men > 65 years old [1], is an important problem that warrants intervention in routine medical settings [2]. This is particularly true for patients with HIV (PWH) given that at-risk levels of alcohol use may interfere with achievement of HIV viral suppression [3], increase risk of morbidity and mortality [4–6], and lead to risk behaviors and ongoing HIV transmission [7]. Accordingly, guidelines recommend that patients with at-risk alcohol use, including those with HIV, should receive brief interventions with subsequent treatment to reduce their alcohol use [8, 9] and integrated with HIV care [10].

Despite its potential to improve individual and public health, brief intervention with subsequent indicated treatment is inconsistently delivered to PWH [11], and, to date, only a limited number of studies have been specifically designed to address unhealthy alcohol use (defined as the spectrum of alcohol use including at-risk drinking and alcohol use disorder) among PWH [12–17]. These studies have generally focused on evaluation of a specific medication (i.e., naltrexone) [12] or behavioral intervention [13–16] and some focused on a specific patient population (e.g., women, men who have sex with men). None of these studies offered a comprehensive package that allowed evaluation of initial patient response to a lower intensity intervention prior to adding additional services. Such “stepped care models” allow for tailoring of treatment based on patient response while employing multidisciplinary team members as needed to deliver specific components of care to maximize resource allocation. Stepped care models have been successfully applied to a variety of medical conditions (e.g., depression, hypertension, chronic pain) [18, 19] addressed in routine medical settings, but rarely applied to address alcohol use [20] and specifically among PWH [13]. We have recently reported the benefits of stepped alcohol treatment for PWH who have alcohol use disorder (as defined by the Diagnostic and Statistical Manual criteria to identify individuals with loss of control and adverse consequences from alcohol use) or lower levels of alcohol use in the presence of liver disease [21, 22]. Notably, this model, because of its approach involving integration of alcohol treatment into routine medical settings with stepped care and demonstrated impact on improving outcomes among PWH with alcohol use disorder, has been endorsed by the National Institute on Alcohol Abuse and Alcoholism [23, 24]. To our knowledge, no prior studies have examined the impact of stepped alcohol treatment integrated with HIV care to address alcohol and HIV outcomes specifically among PWH with at-risk alcohol use and that explicitly includes components designed to promote behavioral and medication-based treatments as indicated. Thus, the aims of this study were to examine the effectiveness of integrated stepped alcohol treatment (ISAT) versus treatment as usual (TAU) on alcohol use and HIV outcomes among PWH with at-risk alcohol use. We hypothesized that ISAT would be associated with improved drinking and health outcomes compared to TAU, with the primary hypothesis being that ISAT would lead to fewer drinks per week compared with TAU.

## Methods

### Study design and setting

The *Starting Treatment for Ethanol in Primary Care (STEP) At-Risk Alcohol Use Trial* was conducted as part of 3 parallel trials addressing different levels of alcohol-related risk in PWH; the two other trials separately enrolled patients who met criteria for moderate alcohol use in the presence of liver disease [21, 25] or alcohol use disorder [22]. The trial was conducted according to standards in the field [26], and the protocol and implementation experiences have been reported [21, 22, 25, 27]. From January 28, 2013 through July 14, 2017, we recruited participants across 5 Veterans Health Administration (VA) Infectious Disease (HIV) Clinics, including in Washington, District of Columbia; Atlanta, Georgia; Brooklyn/Manhattan, New York; and Dallas and Houston, Texas, to participate in the *STEP At-Risk Alcohol Use Trial.*

Patients, the majority of whom were not seeking treatment for their alcohol use, were recruited into the study using a multi-pronged approach including (1) routine or research coordinator-delivered screening with the Alcohol Use Disorder Identification Test-Consumption (AUDIT-C); (2) clinician-referral; (3) patient self-referral; and (4) a list of potentially-eligible patients generated from the medical record.

### Participants

**Fig. 1:**
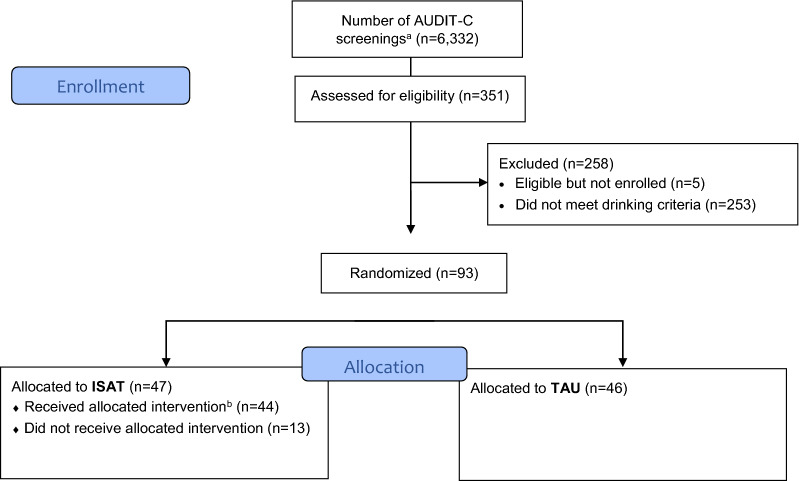
Participant flow

Patients were eligible if they met the following criteria: (1) were HIV positive; (2) received care at one of the 5 participating VA HIV Clinics; (3) English speaking and were able to provide written informed consent; and (4) reported alcohol consumption consistent with 14 or more drinks per week or 4 or more per occasion in men younger than or equal to 65 years old or 7 or more drinks per week or 3 or more drinks per occasion in women or men older than 65 years old by Timeline Followback [TLFB] [28] (Fig. 1).

Patients were excluded if they met any of the following criteria: (1) did not meet drinking criteria as they drank below at-risk levels or met criteria for alcohol use disorder (by mini-SCID) [29]; (2) were acutely suicidal or with a psychiatric condition that affected their ability to provide informed consent or participate in counseling interventions; (3) were currently enrolled in formal treatment for unhealthy alcohol use, excluding self or mutual-help groups (e.g., Alcoholics Anonymous); (4) had any medical condition(s) that would preclude completing the study or cause harm during the course of the study; or (5) were a pregnant or nursing woman, or woman of child-bearing potential who did not agree to use a reliable form of birth control. Since abstinence is recommended during pregnancy and specialty care might be required to achieve this goal, this final criterion was put in place to avoid randomizing pregnant women to treatment as usual.

Participants provided written and informed consent and were reimbursed $25 for baseline assessments and $50 for follow-up assessments. The study was registered at www.clinicaltrials.gov (ClinicalTrials.gov Identifier: NCT01410123).

### Treatment conditions

Eligible and consented patients were randomized to ISAT versus TAU. Regardless of treatment group, participants could receive any non-study services recommended by VA clinicians.

### Integrated stepped alcohol treatment

ISAT interventions were stepped up at pre-defined time points based on a priori criteria and delivered over the course of a 24 week period. Because this was an effectiveness trial, neither patients nor clinicians were specifically incentivized to attend or complete sessions as part of ISAT. ISAT was provided by VA clinicians, including social workers, psychologists and addiction psychiatrists, and whenever possible occurred in the HIV clinics (i.e., co-located), where patients received their routine HIV care.

#### Step 1

Step 1 consisted of a brief psychosocial intervention, the Brief Negotiated Interview (BNI) delivered by an onsite social worker. This manual-guided brief intervention is based upon principles of motivational interviewing and the stages of change model of behavior change. The BNI has demonstrated efficacy in decreasing alcohol use in patients with at-risk drinking [30]. For this trial, the content was modified to address HIV and hepatitis C virus (HCV). The main goals of the session, designed to be 15–20 min long, were to: (1) decrease participant ambivalence to reduce alcohol use by reviewing the participant’s perceptions regarding pros and cons of alcohol use and providing tailored feedback regarding the impact of alcohol on the participant’s medical conditions using the *STEP Trials Feedback Form* and (2) negotiate strategies for change based on the participant’s readiness to change. Participants were also referred to web-based resources for help. Modeled after Project TREAT [31], a telephone booster designed to be 15–20 min in duration occurred 2 weeks after the BNI session. This was also conducted by the social worker and, following a similar structure as the BNI, was designed to review participant progress and challenges towards meeting their drinking goals.

#### Step 2

At the week 4 research assessment, those exceeding at-risk thresholds for alcohol use by TLFB [28] during the prior 14 days were advanced to Step 2, which provided 4 sessions of psychologist-delivered Motivational Enhancement Therapy (MET). MET sessions, scheduled every other week over the course of 6 weeks, were manual-guided with content tailored to PWH [32]. Grounded in motivational interviewing and the stages of change model for behavior change, the psychologists employed reflective listening to help elicit participant-centered reasons to decrease their alcohol use; promoted skill-building as indicated; provided individual-level feedback regarding the potential impact of alcohol on the participant’s health (e.g., increased liver function tests); and offered web-based resources for self-help.

#### Step 3

At week 12, those who were advanced to Step 2 and who continued to exceed at-risk thresholds based on alcohol consumption during the prior 14 days by TLFB, were advanced to Step 3. Step 3 included Addiction Psychiatrist-delivered Addiction Physician Management (APM) with an emphasis on consideration of medications to decrease alcohol use with medical management, consistent with the approach used to provide buprenorphine for treatment of opioid use disorder in HIV treatment settings [33, 34]. Following an initial assessment visit, subsequent visits were scheduled weekly for 2 weeks, every other week for 4 weeks and then monthly for a total of five visits.

### Treatment as usual

As part of recommended care in the VA, for every patient followed in a primary care clinic, including HIV clinics, clinicians are prompted to screen patients annually with an AUDIT-C via a clinical reminder. This reminder includes prompts to the clinician to conduct brief interventions or referral to addiction treatment as indicated [35]. In addition, study participants received a health handout that includes advice about drinking in the context of general health advice (e.g., smoking cessation, exercise) [25].

### Assignment of treatment

We used a web-based clinical trial management system [36] to randomize patients in a 1:1 ratio to ISAT or TAU stratified by site. The randomization sequence was concealed. Blinding of patients, clinicians or research assistants following randomization was not possible.

### Monitoring intervention fidelity and adherence

After initial training of social workers, psychologists and psychiatrists, the study team offered ongoing supervision and monitoring by teleconferences held every 1 to 2 months; provided structured encounter forms to guide intervention sessions; and conducted 2 site visits per site. BNI and MET sessions were digitally recorded and a subset were reviewed with feedback provided by a study psychologist. We tracked the number of completed sessions and the session duration. VA-based pharmacy data were used to assess prescription of Food and Drug Administration (FDA) (i.e., disulfiram, acamprosate and naltrexone) [37] and non-FDA (i.e. topiramate, baclofen and gabapentin) [38] approved medications used to treat alcohol use disorder in the 6 months prior to randomization and through week 52.

### Outcomes

The primary effectiveness outcome was the mean number of drinks per week over the past 30 days at week 24 assessed by TLFB among both ISAT and TAU groups. To assess receipt of the intervention, we determined the proportion of participants who completed ISAT sessions and receipt of alcohol treatment medications.

Secondary drinking outcomes at week 24 and based on the past 30 days by TLFB included the proportion of participants with no heavy drinking days (defined as the absence of any heavy drinking days in the past 30 days, where a heavy drinking days is defined for men ≥ 5 drinks per day and for women as ≥ 4 drinks per day), mean number of drinks per drinking day, and percent of days abstinent; and phosphatidylethanol (PEth) blood levels (an alcohol biomarker that reflects past 21 days of alcohol consumption, with higher levels associated with greater quantities of alcohol use and values of < 8 ng/mL consistent with abstinence or near abstinence) [39]. We also assessed biomarkers based on data collected on the same day or closest to assessments impacted either directly and/or indirectly by alcohol use including: the VACS Index score (validated measure of morbidity and mortality, where higher scores are associated with increasing mortality risk) [4]; and undetectable plasma HIV viral load (HIV RNA < 50 copies/mL). The VACS Index score is created by summing points for age, indicators of HIV disease severity (CD4 cell count, HIV viral load), general indicators of organ system injury (by hemoglobin, estimated glomerular filtration rate, and FIB-4) and presence of hepatitis C virus co-infection. Each five-point increment is associated with an approximately 20% increase in 5 year mortality risk [40] and the VACS Index score varies based on alcohol use [41, 42].

We additionally assessed durability of the intervention by examining outcomes at week 52 (except for PEth, which was only collected at baseline and week 24). PEth was not used to determine study eligibility nor did clinicians or the coordinating center monitor PEth values during the study. Receipt of VA-based outpatient and inpatient addiction treatment services as well as all-cause emergency department visits or hospitalizations were assessed by electronic medical record (EMR) data during the 180 day period prior to baseline, week 24 and week 52, respectively.

### Sample size calculations and statistical analysis

To detect a decrease of 5 drinks per week above the expected decrease of 6.7 drinks per week in those randomized to treatment as usual, a sample of size of 108 participants in each group was needed to have 80% power at the two sided 0.05 significance level [30]. Given an anticipated 15% dropout rate, the target enrollment was 254. With a total of 93 participants, we had 80% power to detect a difference of 7.6 drinks per week in the ISAT group vs. TAU group. (1) We used descriptive statistics to compare baseline characteristics of the treatment groups, report attendance at scheduled intervention visits, proportion receiving treatment medications, and session duration.

Our primary analysis was based on intention-to-treat (ITT) analysis, including all participants in the group to which they were randomized. We defined a patient lost to follow-up if they did not have any assessment at week 24 and afterwards through to week 52. We used linear mixed-effects models to assess: (2) number of drinks per week, (3) number of drinks per drinking day, (4) percent of days abstinent, and (5) VACS Index, with the assumption that missing data occurred at random. Analyses included fixed effects for intervention (ISAT vs. treatment as usual), time (4, 12, 24 and 52 weeks), and the interaction of the intervention with time. Additional fixed effects include the baseline covariates of baseline outcome level, VACS Index score, and site. We included random intercept and time effects for each participant with an unstructured covariance pattern for serial correlation, and present data for the primary outcome as least squares means with 95% confidence intervals (CI). We used linear contrasts to estimate intervention group differences and 95% CIs at week 24 (primary outcome) and week 52. (6) We used linear regression analyses to compare 24 week differences in PEth levels. For binary outcomes, we used generalized linear mixed-effects models with the logit link function. (7) In sensitivity analyses, focused on the primary outcome, we excluded participants with a baseline PEth level < 8 ng/mL reflecting those with minimal to no alcohol use and (8) separately adjusted for baseline heavy drinking given baseline differences between treatment groups.

(9) We did post-hoc adherence adjusted analyses, in which we adjusted for intervention adherence to determine the effect of ISAT that would have been observed if all participants maintained an adequate level of intervention adherence. We used a marginal structural model approach that employs inverse probability weights based on an individual’s propensity to adherence throughout the study [43]. This approach creates a pseudopopulation that removes that confounding effects of adherence. The adequate level of compliance was chosen to be attendance of at least 30% of expected ISAT visits. Stabilized probability weights for less than 30% adherence to ISAT interventions were created from pooled logistic regression across each time period (i.e., weeks 4, 12 and 24) with baseline (age, number of drinks per week, race, site, HIV viral load, other substance use, education, and employment) and time-varying (current and previous number of drinks per week) covariates. The marginal structural model was then implemented by weighted generalized estimating equations (10) We additionally conducted a post-hoc responder analysis and used bivariate analyses to examine baseline sociodemographic and clinical characteristics of individuals across both groups who reduced their average drinks per week by 5 or more at week 24. Then including variables significant in the bivariate analyses as well as treatment group, we created a multivariable model. All analyses involved two-tailed tests of significance and were done using SAS version 9.4 (SAS Institute, Cary, NC, USA).

## Results

### Participant flow

Out of 351 patients who met eligibility criteria for any of the *STEP Trials*, 93 were enrolled into the at-risk drinking trial and randomized (Fig. 1). Despite a multi-pronged approach [25], we only recruited 37% of the target population (n = 93/254). Among the 93 randomized participants, 81 (87%) completed the study (i.e., not lost to follow-up), with 85 (91%) providing data at week 4, 76 (82%) providing data at week 12, 79 (85%) providing data at week 24 and 60 (65%) providing data at week 52.

### Baseline demographic and clinical characteristics

The baseline sociodemographic and behavioral characteristics of participants randomized to ISAT and TAU did not differ (Table 1).

**Table 1:** Participant baseline demographic and clinical characteristics

Characteristic	No. (%)		*p* value
	Integrated stepped alcohol treatment (n = 47)	Treatment as usual (n = 46)	
Men	44 (93.6)	45 (97.8)	0.62
Race			1.00
White	9 (19.2)	9 (19.6)	
Black	37 (78.7)	36 (78.3)	
Other	1 (2.1)	1 (2.2)	
Hispanic	2 (4.4)	4 (8.7)	0.43
Age, mean (SD), y	59.1 (9.6)	56.5 (9.8)	0.19
Education			0.94
High school or less	17 (36.2)	17 (37.0)	
> High school	30 (63.8)	29 (63.0)	
Married or domestic partner	11 (23.4)	7 (15.2)	0.85
Employment status^a^			0.90
Employed	17 (36.2)	19 (41.3)	
Retired/disability	21 (44.7)	19 (41.3)	
Unemployed or unable to work	8 (17.0)	7 (15.2)	
Controlled environment	1 (2.1)	0 (0)	
Student	0 (0)	1 (2.2)	
AUDIT-C score, mean (SD)^b^	5.45 (2.26)	5.50 (2.38)	0.91
Other substance use, past 30 days^c^			
Smoke cigarettes	24 (51.1)	19 (43.2)	0.45
Cannabis	9 (19.2)	11 (23.9)	0.58
Cocaine	2 (4.3)	3 (6.5)	0.68
Heroin	0 (0)	0 (0)	NA
Prescription opioids	1 (2.1)	3 (6.5)	0.36
Comorbid conditions and biomarkers			
Hepatitis C co-infection^d^	12 (25.5)	13 (28.3)	0.77
FIB-4 score > 1.45^e, f^	36 (76.6)	32 (69.6)	0.44
Depressive symptoms^g^	7 (14.9)	7 (15.2)	0.97
HIV related measures			
VACS Index, median (range)^f,h^	33 (0,93)	28 (6,68)	0.34
Detectable HIV viral load^f,i^	17 (36.2)	15 (32.6)	0.72
CD4 cell count, cells/mm^3^, median (range)^f^	542 (112,1427)	537 (109,1255)	0.91

^a^Employment status, employment during past 3 years: assessed based on the Addiction Severity Index Lite-CF[54]

^b^Alcohol Use Disorders Identification Test-Consumption (AUDIT-C) scores range from 0 to 12

^c^Other substance use, past 30 days: assessed based on item “Do you know smoke cigarettes (as of 1 month ago)?” and the Addiction Severity Index Lite-CF[54]

^d^Hepatitis C coinfection status—based on positive antibody and detectable HCV RNA viral load

^e^FIB-4 score—a noninvasive measure of liver fibrosis calculated based on aspartate aminotransferase, alanine aminotransferase, and platelets with scores greater than 1.45 concerning for liver fibrosis

^f^Laboratory testing performed within 30 days prior to randomization date

^g^Depressive symptoms determined using the Patient Health Questionnaire (PHQ)-9 with score > 9 defined as having depressive symptoms[55]

^h^VACS index—validated measure of morbidity and mortality risk [40]

^i^Detectable HIV viral load—defined as ≥ 50 copies/mL

### ISAT intervention receipt

**Fig. 2:**
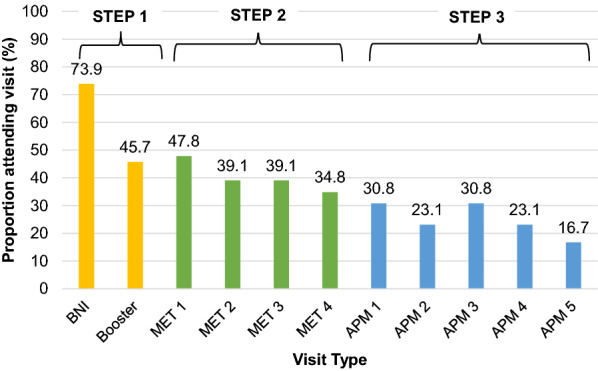
Visit attendance among those randomized to integrated stepped alcohol treatment. Denominator is among those eligible for the intervention: Step 1: n = 46; Step 2: n = 23; Step 3: n = 13 for APM1-4, n = 12 for APM 5. *BNI*  Brief Negotiated Interview, *MET* Motivational Enhancement Therapy, *APM* Addiction Physician Management

Regarding Step 1, 74% received the BNI and 46% received the telephone booster. 50% (23/46) met criteria for advancing to Step 2, and 13 of 23 in Step 2 (57%) met criteria for advancing to Step 3. Among those advanced to Step 2, 48% attended the first visit while 35% attended the fourth visit (Fig. 2). Among those advanced to Step 3, 31% attended the first APM visit, while 17% attended the 5th visit. Across the four MET sessions, the median duration was 30 min, ranging from 9 to 60 min; across the five APM sessions, the median duration was 30 min, ranging from 10 to 60 min. There were no observed differences by treatment group for receipt of alcohol treatment medications (Table 2).

**Table 2:** Past 6 month receipt of alcohol treatment medications at baseline and follow-up by treatment group

Medication, n (%)	Integrated stepped alcohol treatment (n = 47)	Treatment as usual (n = 46)	*p* value
Any alcohol treatment medication^a^			
Baseline	5 (10.6)	10 (21.7)	0.15
Week 24	8 (17.0)	11 (23.9)	0.41
Week 52	5 (10.6)	6 (13.0)	0.72
Disulfiram			
Baseline	0 (0)	0 (0)	NA
Week 24	0 (0)	0 (0)	NA
Week 52	0 (0)	0 (0)	NA
Acamprosate			
Baseline	0 (0)	0 (0)	NA
Week 24	0 (0)	0 (0)	NA
Week 52	0 (0)	0 (0)	NA
Naltrexone			
Baseline	0 (0)	0 (0)	NA
Week 24	1 (2.1)	1 (2.2)	0.99
Week 52	1 (2.1)	0 (0)	0.99
Topiramate			
Baseline	0 (0)	0 (0)	NA
Week 24	0 (0)	0 (0)	NA
Week 52	0 (0)	0 (0)	NA
Baclofen			
Baseline	1 (2.1)	2 (4.4)	0.62
Week 24	2 (4.3)	2 (4.4)	0.99
Week 52	2 (4.3)	1 (2.2)	0.99
Gabapentin			
Baseline	4 (8.5)	8 (17.4)	0.20
Week 24	6 (12.8)	8 (17.4)	0.53
Week 52	4 (8.5)	5 (10.9)	0.74

^a^Based on receipt of disulfiram, acamprosate, naltrexone, topiramate, baclofen and/or gabapentin

### Alcohol consumption outcomes

#### Primary outcome, past 30 day self-reported

**Fig. 3:**
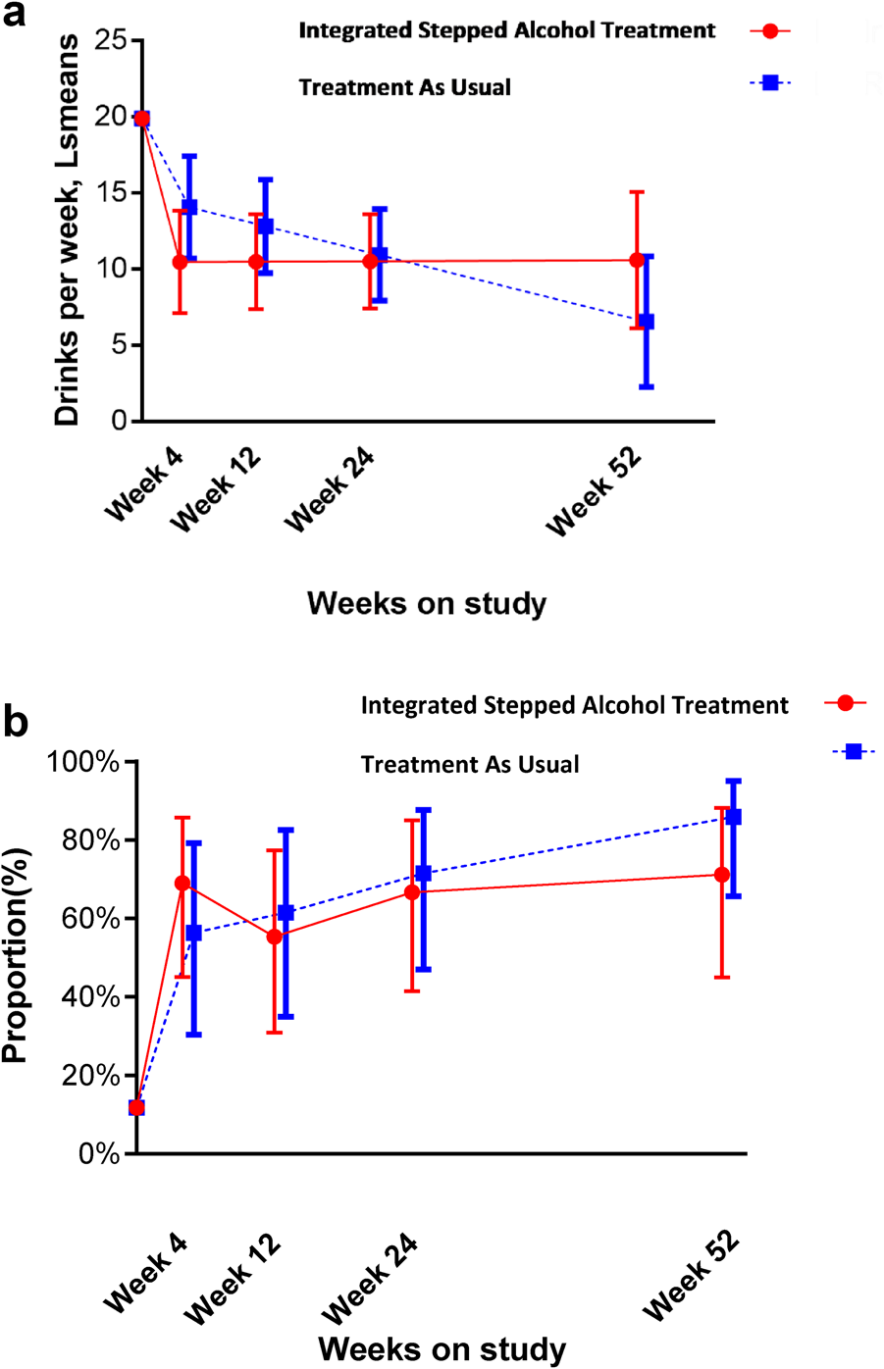
Drinking outcomes. **a** Drinks per week over the past 30 days; **b** Proportion of participants with no heavy drinking days over the past 30 days. Data points are least square means or proportions with whiskers denoting 95% CIs

Both groups had evidence of decreased alcohol use (Fig. 3a, Additional file 1: Table S1). At week 24 (primary outcome), while findings favored ISAT, we did not see a significant difference between the ISAT and TAU groups in number of drinks per week over the past 30 days [least square means (Ls mean) (95% CI) = 8.8 vs. 10.6; adjusted mean difference (AMD) (95% CI) = − 0.4 (− 3.9, 3.0)]. Similarly, no differences between groups were observed at week 52.

## Other alcohol consumption outcomes

The proportion of participants with no heavy drinking days was not significantly different among those randomized to ISAT compared to TAU at week 24 [47 vs. 43%, adjusted odds ratio [AOR] (95% CI) = 0.8 (0.2, 2.6)] and at week 52 [52 vs. 58%, AOR (95% CI) = 0.4 (0.1, 1.5)] (Fig. 3b, Additional file 1: Table S1). The percentage of days abstinent did not differ among those randomized to ISAT compared to TAU at week 24 (LSmean = 60 vs. 66%), AMD [95% CI] = − 2 [− 10, 6] and at week 52 [LSmean = 54 vs. 67%, AMD = − 11 (− 25, 3)]. At week 24, PEth concentrations did not differ between participants in the ISAT and TAU groups.

### HIV biomarkers

At week 24, participants randomized to ISAT did not differ in VACS Index scores compared to those randomized to TAU [LSmean = 34 vs. 32; AMD (95% CI) = -0.4 (− 4.7, 4.1)]. Findings were consistent at week 52. The proportion with an undetectable HIV viral load also did not differ among those randomized to ISAT compared to those randomized TAU at week 24 [76 vs. 84%, AOR (95% CI) = 0.6 (0.1, 2.8)] or week 52 [71 vs. 77%, AOR (95% CI) = 0.8 (0.2, 3.8)].

### Healthcare use

At 24 weeks, the ISAT and TAU groups did not differ on receipt of outpatient alcohol treatment, inpatient alcohol treatment, Emergency Department visit number or hospitalizations (Table 3). Findings were consistent at week 52.

**Table 3:** Past 6 month treatment services, emergency department visits and hospitalizations at baseline and follow-up by treatment group

	Integrated stepped alcohol treatment (N = 47)	Treatment as usual (N = 46)	*p* value
Any outpatient alcohol treatment^a, b^			
Baseline	0 (0)	0 (0)	NA
Week 24	16 (34.0)	12 (26.1)	0.40
Week 52	9 (19.2)	12 (26.1)	0.42
Any inpatient alcohol treatment^a, b^			
Baseline	0 (0)	2 (4.4)	0.24
Week 24	0 (0)	1 (2.2)	0.49
Week 52	0 (0)	2 (4.4)	0.24
Emergency department visits^b^			
Baseline	6 (12.8)	12 (26.1)	0.10
Week 24	12 (25.5)	13 (28.3)	0.77
Week 52	11 (23.4)	15 (32.6)	0.32
Hospitalization^b^			
Baseline	2 (4.3)	7 (15.2)	0.09
Week 24	6 (12.8)	8 (17.4)	0.53
Week 52	9 (19.2)	9 (19.6)	0.96

^a^Based on presence of an alcohol or drug related ICD-9 or ICD-10 code at any time and, for outpatient services, if they had a SUD clinic stop code or CPT code; for inpatient services, included if they had a SUD bed section stop code or ICD-9 or ICD-10 procedure code

^b^Assessed using VA electronic medical record data and during the 180 day period prior to baseline, week 24, and week 52, respectively

### Sensitivity and post-hoc analyses

In sensitivity analyses, excluding those with a baseline PEth < 8 ng/mL, we did not detect a difference between the ISAT and TAU groups in number of drinks per week over the past 30 days [least square means (Ls mean) (95% CI) = 11.0 vs. 11.5; adjusted mean difference (AMD) (95% CI) = − 5.9 (− 4.3, 3.1)].

Adjusting for baseline non-heavy drinking, we did not see a difference between the ISAT and TAU groups in number of drinks per week over the past 30 days [least square means (Ls mean) (95% CI) = 6.6 vs. 9.5; adjusted mean difference (AMD) (95% CI) = − 2.9 (− 9.1, 3.3)].

In the *post-hoc* per protocol analysis adjusted for intervention adherence, ISAT participants who completed at least 30% of interventions visits [n = 31 (66%)] did not differ in number of drinks per week compared to TAU participants [Lsmean = 8.3 vs. 7.5, AMD (95% CI) = 0.8 (− 3.5, 5.0)]. In the responder analysis, among the 81 participants included, 46 (56%) met criteria for response; baseline characteristics associated with a reduction of an average of 5 or more drinks per week at week 24 from baseline include baseline drinks per week and smoking status (Additional file 1: Table S2). In multivariate analyses adjusted for treatment group, average drinks per week [AOR (95% CI) = 1.1 (1.1, 1.3)] and smoking status [AOR (95% CI) = 4.9 (1.3, 18.1)] remained associated with response such that for every increase in average drinks per week, there was a 10% increased likelihood of response and participants who did not smoke had a fivefold increased odds of response, respectively.

## Discussion

The *STEP At-Risk Trial,* which aimed to evaluate the effectiveness of ISAT compared to TAU on alcohol use and HIV outcomes among PWH with recent (i.e., past 30 days) at-risk alcohol use, generated several important findings. First, ISAT was a feasible model for improving delivery of evidence-based interventions to PWH with at-risk alcohol use with 16–74% completing a given intervention visit, including over one third who completed visits associated with Step 1 and Step 2. Second, given that 50% of participants randomized to ISAT were “stepped up” to step 2 and 57% were then advanced to step 3, our findings demonstrate the utility of a stepped care model for addressing at-risk alcohol use in this population. Third, engaging PWH with at-risk levels of alcohol use in alcohol-related care is a challenge. Lastly, we found that participants who smoke were less likely to decrease their alcohol use regardless of intervention group.

Our findings extend the existing literature focused on developing interventions to address at-risk alcohol use among PWH [17]. It is notable that clinic-based interventions to address at-risk alcohol use among PWH are relatively scarce. Based on a systematic review of the literature and meta-analysis, Scott-Sheldon and colleagues reported in 2017 that there were only 21 studies that reported on individual-level interventions designed to address alcohol use among PWH, 71% of which were clinic-based [17]. Consistent with our approach, studies that targeted alcohol alone (vs. multiple HIV-related behaviors) and that were clinic-based (vs. recruiting from other/mixed settings), were found to be more successful at decreasing alcohol use[14–17], though these effects may vary based on baseline level of motivation and intervention strategy[13]. Building on this literature and our experiences evaluating ISAT among PWH with higher and lower levels of alcohol use[21, 22], the current study provides additional support for approaches that include MI-based interventions that target alcohol use in clinic-based settings to promote alcohol reduction among PWH. Specifically, we found that some PWH – even when not specifically incentivized to do so—will attend visits to address their alcohol use. In addition, we found that graded interventions and those incorporating MI are appropriate; 50% of participants met criteria for MET after an initial brief intervention with booster and nearly 45% responded to these MET sessions and did not meet criteria for further “stepping up.” The stepped care model served to enhance receipt of delivery of evidence-based interventions to optimize resources given its sequential nature. This stepped care approach is relevant for primary care based settings given the potential to incorporate multidisciplinary team members to deliver specific components, such as the brief intervention [44]. Whether this model and treatment receipt translates into changes in alcohol use and HIV-related outcomes for PWH with at-risk alcohol use, however, is not yet clear. In addition, our findings should be interpreted in the context of recent VA-based analyses demonstrating that brief intervention is associated with decreased likelihood of receipt of specialty addiction services [45]; however, it is an empirical question how these findings generalize to PWH seen in HIV clinics, where mental health services are often embedded. In addition, the recognized harmful effects of concurrent alcohol and tobacco use [46, 47] and our findings that current smoking was negatively associated with alcohol reductions over time together reinforce the need for evaluation of targeted interventions to address these commonly co-occurring, mutually-reinforcing behaviors [48]. Stepped care interventions, including behavioral interventions (e.g., contingency management or cognitive behavioral therapy) in combination with medications, such as varenicline, merit further evaluation.

Our study should be interpreted in the context of its limitations. First, despite a multi-pronged approach to enroll participants, we were unable to meet recruitment targets and were underpowered to evaluate the impact of our intervention on participant-level outcomes. We believe this relates to low prioritization to address alcohol use among this patient population in the context of at-risk alcohol use [49–51], underlying depressive symptoms, as well as challenges with logistics associated with study participation (e.g., travel, childcare responsibilities, employment) as has been observed in other alcohol intervention studies for PWH [52]. Formative evaluation to better understand why patients do and do not opt to participate in an alcohol intervention study and how these populations differs may be useful. Meanwhile, novel strategies, including use of incentives and remote methods for intervention delivery, to promote patient engagement in alcohol interventions may be useful and are actively being evaluated [53]. Second, we assumed missing data were missing at random; however, we acknowledge that there is no method to validate this assumption and that a missing not at random (MNAR) process may have biased results whereby participants with missing data may have been more likely to have higher levels of alcohol use. Given that more patients in the ISAT group were lost to follow-up, this may have biased us towards the null hypothesis. Third, our findings may not be generalizable to women or patients receiving care outside the VA. Fourth, we relied on research assistants to identify potentially eligible participants and to determine whether they meet criteria for being stepped up. Future study is needed to determine how this might be translated into routine clinical practice. Lastly, our control condition, TAU, may reflect a higher level of alcohol-related care than is routine in other HIV treatment settings, given routine implementation of AUDIT-C screening and electronic health record prompts to trigger brief intervention when indicated.

## Conclusions

ISAT may hold promise as a model to promote alcohol-related care among PWH across the spectrum of alcohol use. Future studies are needed to enhance patient-level engagement to initiate and remain in alcohol interventions and bolster the impact of ISAT on patient-level outcomes among PWH with at-risk alcohol use.

## Supplementary information

**Additional file 1: Table S1** Drinking and HIV-related outcomes by treatment group. **Table S2** Participant baseline demographic and clinical characteristics associated with treatment response.

## Data Availability

With written requests and after review and approval by the Principal Investigators (DAF and AC) and the Veterans Aging Cohort Study Team, a data dictionary defining each field in the analytic data set and de-identified individual participant data will be made available after findings of the main analyses have been published. Details of the study protocol have been previously published [25].

## Abbreviations

AMD: Adjusted means difference
AOR: Adjusted odds ratio
APM: Addiction Physician Management
AUDIT-C: Alcohol Use Disorders Identification Test-Consumption
BNI: Brief Negotiated Interview
CI: Confidence interval
EMR: Electronic medical record
FDA: Food and Drug Administration
HCV: Hepatitis C virus
HIV: Human Immunodeficiency Virus
ISAT: Integrated stepped alcohol treatment
ITT: Intention to treat
LS mean: Least square mean
MET: Motivational Enhancement Therapy
PEth: Phosphatidylethanol
PWH: Patients with HIV
RNA: Ribonucleic acid
SAS: Statistical analysis system
SCID: Structured Clinical Interview for DSM
STEP: Starting Treatment for Ethanol in Primary Care
TAU: Treatment as usual
TLFB: Timeline Followback
TREAT: Trial for early alcohol treatment
VACS: Veterans Aging Cohort Study
VA: Veterans Health Administration
