# Supplementary material for: Integrated stepped alcohol treatment for patients with HIV and at-risk alcohol use: a randomized trial
Source: Addict Sci Clin Pract. 2020 Jul 29;15:28. doi: 10.1186/s13722-020-00200-y (PMC7388231; doi:10.1186/s13722-020-00200-y)
Supplement: Supplementary file 1 — Additional file 1: Table S1 Drinking and HIV-related outcomes by treatment group. Table S2 Participant baseline demographic and clinical characteristics associated with treatment response. [file 13722_2020_200_MOESM1_ESM.docx]

**Appendix Table 1. Drinking and HIV-related outcomes by treatment group**

| **Outcome** | **Integrated Stepped Alcohol Treatment, n=47** | **Treatment as Usual,**  **n=46** | **Adjusted Treatment Effect**  **(95% CI)**  **ISAT vs. TAU^a,b^** | ***P* value^b^** |
| --- | --- | --- | --- | --- |
| **Drinks per week, Lsmean (SD)** | | | | |
| Baseline | 17.0 (10.9) | 22.8 (28.8) | NA | NA |
| Week 24 | 8.8 (8.6) | 10.6 (14.8) | -0.4 (-3.9, 3.0) |  |
| Week 52 | 10.6 (12.3) | 11.0 (19.6) | 4.0 (-1.6, 9.7) |  |
| **No heavy drinking days, n (%)^c^** | | | | |
| Baseline | 10/47 (21.3) | 1/46 (2.2) | NA | NA |
| Week 24 | 16/34 (47.1) | 19/44 (43.2) | 0.80 (0.2, 2.6) | 0.71 |
| Week 52 | 15/29 (51.7) | 18/31 (58.1) | 0.4 (0.1, 1.5) | 0.19 |
| **Drinks per drinking day, Lsmean (SD)** | | | | |
| Baseline | 5.6 (3.4) | 6.2 (3.8) | NA | NA |
| Week 24 | 3.6 (1.9) | 4.1 (3.9) | -0.4 (-1.4, 0.7) | 0.47 |
| Week 52 | 4.0 (3.4) | 4.2 (3.6) | 0.2 (-1.4, 1.7) | 0.82 |
| **Percentage of days abstinent, Lsmean (SD)** | | | | |
| Baseline | 44.3 (35.5) | 49.2 (33.3) | NA | NA |
| Week 24 | 60.0 (32.1) | 65.6 (32.3) | -1.9% (-10.2%, 6.3%) | 0.64 |
| Week 52 | 53.9 (39.5) | 67.0 (33.7) | -11.2% (-25.0%, 2.7%) | 0.11 |
| **PEth, Lsmean (SD)^d^** | | | | |
| Baseline | 112.0 (143.3) | 135.4 (198.8) | NA | NA |
| Week 24 | 148.8 (174.9) | 116.8 (202.9) | 48.3 (-20.4, 116.9) | 0.16 |
| **VACS Index, Lsmean (SD)^e^** | | | | |
| Baseline | 34.6 (20.4) | 30.4 (16.4) | NA | NA |
| Week 24 | 33.8 (16.9) | 31.6 (18.5) | -0.4 (-4.7,4.1) | 0.87 |
| Week 52 | 32.9 (16.8) | 31.1 (19.9) | -1.5 (-7.7, 4.7) | 0.62 |
| **Undetectable HIV viral load, n (%)^f^** | | | | |
| Baseline | 30/47 (63.8) | 31/46 (67.4) | NA |  |
| Week 24 | 22/29 (75.9) | 27/32 (84.4) | 0.6 (0.1, 2.8) | 0.51 |
| Week 52 | 20/28 (71.4) | 20/26 (76.9) | 0.8 (0.2, 3.8) | 0.83 |

a. Unadjusted estimates

b. Estimates based on results from linear mixed effects models for continuous outcomes and generalized linear mixed effects models for binary outcomes. For binary variables, treatment effects are expressed as an adjusted odds ratio.

c. Values adjusted for baseline assessment, baseline VACS Index and site (except PEth focused analyses only adjusted for baseline PEth and VACS index)

d. No heavy drinking defined as the absence of any heavy drinking days in the past 30 days, where a heavy drinking days is defined for men >5 drinks per day and for women as >4 drinks per day.

e. PEth = phosphatidylethanol, an alcohol biomarker reflecting alcohol use over the past 21 days and higher scores are associated with higher levels of alcohol use. PEth <8ng/mL consistent with abstinence or near abstinence(39).

f. VACS Index is a validated measure of morbidity and mortality based on age, HIV biomarkers (CD4 cell count, HIV viral load), hemoglobin, FIB-4 score (a measure of liver fibrosis calculated based on aspartate and alanine aminotransferase and platelet count), creatinine and hepatitis C status. Scores range from 0 to 164; each 5 point increase is associated with a 20% increase in 5 year mortality risk(4, 40).

g. Undetectable HIV viral load was based on the limit of detection and defined as <50 copies/mL.

**Appendix Table 2. Participant Baseline Demographic and Clinical Characteristics associated with Treatment Response**^a^

|  | **No. (%)** | | **p value** |
| --- | --- | --- | --- |
| **Characteristic** | **Non-responder**  **N=35** | **Responder**  **N=46** |  |
| **Men** | 33 (94.3%) | 45 (97.8%) | 0.58 |
| **Race** |  |  | 0.47 |
| White | 07 (20.0%) | 06 (13.0%) |  |
| Black | 07 (20.0%) | 06 (13.0%) |  |
| Other | 00 (0.0%) | 02 (4.4%) |  |
| **Hispanic** | 03 (8.8%) | 02 (4.4%) | 0.37 |
| **Age**, mean (SD), y | 57.3 (10.7) | 58.1 (7.8) | 0.70 |
| **Education** |  |  | 0.86 |
| High school or less | 13 (37.1%) | 18 (39.1%) |  |
| >High school | 22 (62.9%) | 28 (60.9%) |  |
| **Married or domestic partner** | 6 (7.1%) | 9 (19.6%) | 0.39 |
| **Employment status** |  |  | 0.87 |
| Employed | 13 (37.1%) | 18 (39.1%) |  |
| Retired/disability | 15 (42.9%) | 18 (39.1%) |  |
| Unemployed | 06 (17.1%) | 09 (19.6%) |  |
| Controlled environment | 00 (0.0%) | 01 (2.2%) |  |
| Student | 01 (2.9%) | 00 (0.0%) |  |
| **AUDIT-C score**, mean (SD) | 4.8 (1.9) | 6.0 (2.4) | 0.01* |
| **Drinks per week,** median (range) | 7.6 (1.2 – 28.2) | 22.6 (5.5 – 181.1) | <0.001 |
| **Other substance use, past 30 days** |  |  |  |
| Smoke cigarettes | 22 (62.9%) | 16 (36.4%) | 0.02 |
| Cannabis | 05 (14.3%) | 12 (26.1%) | 0.20 |
| Cocaine | 02 (5.7%) | 03 (6.5%) | 1.00 |
| Heroin | 0 (0.0%) | 0 (0.0%) | N/A |
| Prescription opioids | 01 (2.9%) | 03 (6.5%) | 0.63 |
| **Comorbid conditions and biomarkers** |  |  |  |
| Hepatitis C co-infection | 10 (28.6%) | 14 (30.4%) | 0.86 |
| FIB-4 score> 1.45 | 24 (68.6%) | 36 (78.3%) | 0.32 |
| Depressive symptoms | 05 (14.3%) | 07 (15.2%) | 0.91 |
| **HIV related measures** |  |  |  |
| VACS Index, median (range) | 28.0 (6.0 – 68.0) | 28.5 (0.0 – 93.0) | 0.51 |
| Detectable HIV viral load | 14 (40.0%) | 15 (32.6%) | 0.49 |
| CD4 cell count, cells/mm^3^, median  (range) | 513  (112 – 1386) | 561  (109 – 1427) | 0.83 |

1. Response defined as reduction of 5 or more average drinks per week over the past 30 days at week 24 compared to baseline among participants with TLFB data at week 24.
